# Cortico-Striatal-Thalamic Loop Circuits of the Orbitofrontal Cortex: Promising Therapeutic Targets in Psychiatric Illness

**DOI:** 10.3389/fnsys.2017.00025

**Published:** 2017-04-27

**Authors:** Peter Fettes, Laura Schulze, Jonathan Downar

**Affiliations:** ^1^Institute of Medical Science, University of TorontoToronto, ON, Canada; ^2^Krembil Research Institute, University Health NetworkToronto, ON, Canada; ^3^Department of Psychiatry, University of TorontoToronto, ON, Canada; ^4^MRI-Guided rTMS Clinic, University Health NetworkToronto, ON, Canada

**Keywords:** orbitofrontal cortex (OFC), corticostriatal circuits, psychiatric disease, brain stimulation, research domain criteria (RDoC)

## Abstract

Corticostriatal circuits through the orbitofrontal cortex (OFC) play key roles in complex human behaviors such as evaluation, affect regulation and reward-based decision-making. Importantly, the medial and lateral OFC (mOFC and lOFC) circuits have functionally and anatomically distinct connectivity profiles which differentially contribute to the various aspects of goal-directed behavior. OFC corticostriatal circuits have been consistently implicated across a wide range of psychiatric disorders, including major depressive disorder (MDD), obsessive compulsive disorder (OCD), and substance use disorders (SUDs). Furthermore, psychiatric disorders related to OFC corticostriatal dysfunction can be addressed via conventional and novel neurostimulatory techniques, including deep brain stimulation (DBS), electroconvulsive therapy (ECT), repetitive transcranial magnetic stimulation (rTMS), and transcranial direct current stimulation (tDCS). Such techniques elicit changes in OFC corticostriatal activity, resulting in changes in clinical symptomatology. Here we review the available literature regarding how disturbances in mOFC and lOFC corticostriatal functioning may lead to psychiatric symptomatology in the aforementioned disorders, and how psychiatric treatments may exert their therapeutic effect by rectifying abnormal OFC corticostriatal activity. First, we review the role of OFC corticostriatal circuits in reward-guided learning, decision-making, affect regulation and reappraisal. Second, we discuss the role of OFC corticostriatal circuit dysfunction across a wide range of psychiatric disorders. Third, we review available evidence that the therapeutic mechanisms of various neuromodulation techniques may directly involve rectifying abnormal activity in mOFC and lOFC corticostriatal circuits. Finally, we examine the potential of future applications of therapeutic brain stimulation targeted at OFC circuitry; specifically, the role of OFC brain stimulation in the growing field of individually-tailored therapies and personalized medicine in psychiatry.

## Introduction

The past quarter-century has seen tremendous advances in our understanding of the functions of the frontal lobes of the human brain. However, of the three major surfaces of the frontal lobes—lateral, medial and orbital—it is the latter that is arguably the least well-understood. The orbitofrontal cortex (OFC) is a capacious shelf of gray matter, occupying the entire ventral surface of the frontal lobes, and containing a diverse array of cytoarchitectonic regions whose membership and borders are still a subject of debate among neuroanatomists. As for the functions of these regions, several parallel literatures have developed, each focusing on different hypothetical functions performed by the OFC: the assignment of value (Montague and Berns, 2002), reward and reversal learning (Kringelbach, 2005; Fellows, 2007), reward prediction error and fictive error (Boorman et al., 2013), the generation of affective states (Bechara et al., 2000), emotional reevaluation and reappraisal (Johnstone et al., 2007; Wager et al., 2008), decision-making (McClure et al., 2004), and social cognition, among others (Rushworth et al., 2007; Jonker et al., 2015).

One point of agreement is that the OFC plays a critical role in many of the complex functions that are essential to healthy human cognition, affect regulation and behavior. When these functions are disrupted, psychiatric illnesses may ensue. As our understanding of the neural basis of mental illness steadily improves, the OFC increasingly appears to play an important, and in some cases central, role in the pathophysiology of mood, anxiety, psychotic and other major categories of psychiatric disorder (Drevets, 2007; Nakao et al., 2014; Voon et al., 2015; Cheng et al., 2016). More specifically, an increasingly diverse array of psychiatric symptoms seem to be associated with abnormal functioning in basal ganglia “loop circuits” from the OFC to associated regions of the striatum, pallidum, thalamus, subthalamic nucleus, and midbrain dopaminergic structures (Drevets, 2007; Gorwood, 2008; Ahmari and Dougherty, 2015; Wood and Ahmari, 2015). Obsessive-compulsive disorder (OCD) was perhaps the first psychiatric disorder for which OFC-basal ganglia loop dysfunction was recognized (Baxter et al., 1988). However, it is increasingly clear that such dysfunction also plays a critical role in the pathophysiology of other disorders such as substance use disorders (SUDs) and major depressive disorder (MDD), among others (Drevets, 2007; Goldstein and Volkow, 2011; Volkow et al., 2011).

A lateral orbitofrontal corticostriatal loop circuit was named among one of the five “functionally segregated” basal ganglia loop circuits originally described by Alexander et al. (1986). However, it is now becoming clearer that multiple OFC-basal ganglia loop circuits may exist, each with slightly different roles (Bonelli and Cummings, 2007). Moreover, it is increasingly recognized that frontal lobe loop circuits are not, in fact, sharply segregated but instead have more of a partially-open, partially-closed architecture (Averbeck et al., 2014). As such, it stands to reason that psychiatric disorders may involve dysfunction not merely within an isolated lateral OFC (lOFC) loop, but more diffusely across multiple loops, whose architecture stands partially within and partially outside the OFC proper (e.g., Milad and Rauch, 2012). These loops may, however, map on to distinct clusters of symptoms that exist transdiagnostically across traditional categories of psychiatric disease. These symptom dimensions may correspond reasonably well to the major Research Domain Criteria (RDoC) constructs such as positive valence systems, negative valence systems, and cognitive control systems (Insel, 2014; Dunlop et al., 2016a), each subserved by distinct neural pathways (Webb et al., 2016). Multiple OFC loops, or OFC-interacting loops, may therefore serve as neural substrates for distinct dimensions of psychiatric illness.

From a clinical perspective, the anatomy and function of OFC loops in psychiatric illness is becoming increasingly relevant in the present era, thanks to the advent of new techniques for therapeutic brain stimulation. Unlike conventional psychotherapy or psychopharmacological interventions, brain stimulation treatments are anatomically circumscribed in their targets, so choosing the appropriate anatomical target is essential for treatment success. There is growing evidence that most brain stimulation treatments, from surgically invasive techniques such as deep brain stimulation (DBS) to less invasive techniques such as electroconvulsive therapy (ECT), repetitive transcranial magnetic stimulation (rTMS), or transcranial direct current stimulation (tDCS), exert therapeutic effects by modulating the activity of corticostriatal circuits (Strafella et al., 2001; Takano et al., 2007; Lozano et al., 2008; Bewernick et al., 2010; Chib et al., 2013; Downar et al., 2014; Dunlop et al., 2016b). These techniques have only recently begun to be directed against OFC-striatal targets specifically; however, the available evidence to date suggests that OFC-striatal stimulation may be a promising approach in cases where medications, therapy, or even stimulation of non-OFC targets have failed (Lozano et al., 2008; Bewernick et al., 2010; Nauczyciel et al., 2014; Mondino et al., 2015; Bation et al., 2016; Fettes et al., 2017). OFC-stimulation may therefore offer the potential to enhance remission rates in treatment-resistant illness.

This review article considers the corticostriatal circuitry of the OFC from several different perspectives. First, we will review available evidence about the structural and functional anatomy of the OFC and its associated basal ganglia loop circuits, with a focus on medial and lateral sub-circuits of the classically described lOFC-basal ganglia loop. Next, we will examine the association between abnormal activity within these circuits and specific symptom clusters in psychiatric illnesses, focusing specifically on MDD, OCD, and SUDs. Then, we will consider available evidence on how brain stimulation treatments modulate activity in OFC corticostriatal loop circuits, and how these effects contribute to the therapeutic mechanisms of brain stimulation in psychiatric illness. Finally, we will consider a series of unresolved issues and questions for future study on the role of OFC-striatal circuits in psychiatric disease and its treatment.

## Anatomical Structure of OFC Cortico-Striatal Loop Circuits

### Medial-Lateral Division of the Orbitofrontal Cortex

**Figure 1:**
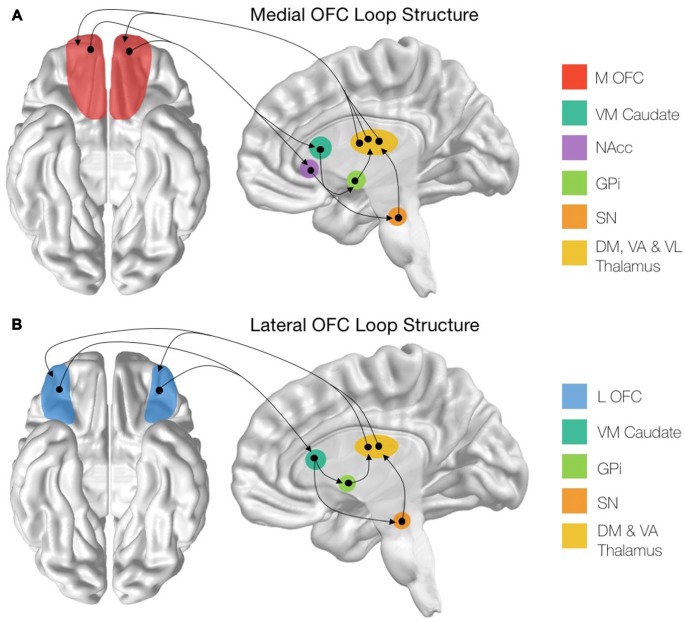
**Structure of the cortico-striatal loops stemming from the orbitofrontal cortex (OFC) subregions. (A)** Schematic showing the entire medial OFC (mOFC) cortico-striatal loop, originating from the mOFC shown in red. **(B)** Schematic showing the entire lateral OFC (lOFC) cortico-striatal loop, originating from the lOFC shown in blue. mOFC, medial orbitofrontal cortex; VM Caudate, ventromedial caudate; NAcc, nucleus accumbens; GPi, globus pallidus interna; SN, substantia nigra; DM Thalamus, dorsomedial thalamic nuclei; VA Thalamus, ventroanterior thalamic nuclei; VL Thalamus, ventrolaterial thalamic nuclei.

The ventral surface of the frontal lobe is often referred to generically as a single region, the OFC. However, there is substantial evidence suggesting that there are cytoarchitectonically distinct subregions within the OFC, each differing greatly with respect to cortical and subcortical anatomical connectivity (Chavis and Pandya, 1976; Cavada et al., 2000; Uylings et al., 2010; Henssen et al., 2016). Specifically, while examining how disturbances in the cortical and subcortical circuits of the OFC lead to the emergence of psychiatric symptomatology, it is important to make a distinction between the OFC’s medial and lateral divisions (Kringelbach and Rolls, 2004; Bonelli and Cummings, 2007; Figure 1).

The anatomical distinction between the medial and lateral OFC (mOFC and lOFC, respectively) is apparent in terms of both micro-structural and macro-structural connectivity. Regarding the former, cytoarchitectonic maps as far back as the seminal atlas of identify distinct subregions in the mOFC and lOFC, characterized by differences in the microscopic appearance (as reviewed in Kringelbach, 2005; Henssen et al., 2016). To summarize, Brodmann areas (BA) 10, 11, and 47 form the lOFC, whereas the caudal part of the mOFC is delineated as BA 25 and 12 (as reviewed in Elliott et al., 2000). Later investigations further subdivided the OFC into additional subregions based on granularity and other staining criteria (Walker, 1940).

**Figure 2:**
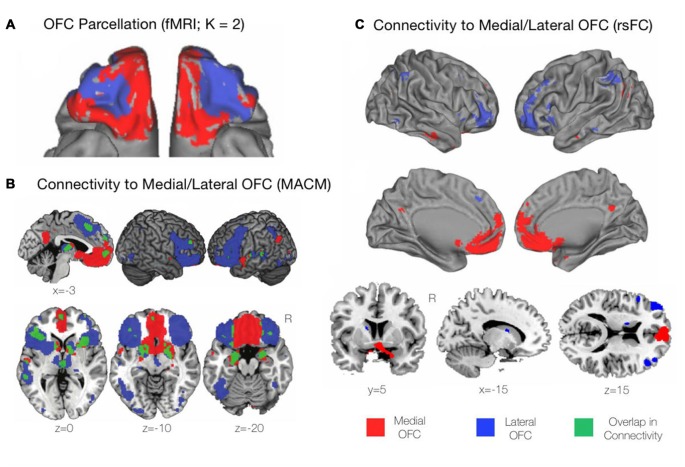
**Functional profiles of the mOFC and lOFC subregions. (A)** A connectivity-based parcellation of the OFC from resting-state functional magnetic resonance imaging (fMRI) revealed distinct medial and lateral subdivisions based on a *K* = 2 cluster solution using *K*-means clustering. Adapted from Kahnt et al. (2012). **(B)** Functional connectivity of the mOFC and lOFC subregions using meta-analytic connectivity modeling (MACM). The mOFC was found to primarily coactivate with regions of the default mode network (DMN), including the ventromedial prefrontal cortex (VMPFC) and the posterior cingulate cortex (PCC). The lOFC coactivated with cognitive control regions including the dorsomedial and dorsolateral prefrontal cortices (DMPFC and DLPFC, respectively), as well regions of the salience network including the bilateral anterior insula and rostral anterior cingulate cortex (rACC). Adapted from Zald et al. (2014). **(C)** Functional connectivity of the mOFC and lOFC subregions using resting-state functional connectivity (rsFC) analyses. As with the findings of the MACM technique described above, the mOFC was functionally connected with regions of the DMN, while the lOFC was functionally connected to cognitive control regions. Adapted from Kahnt et al. (2012).

The medial and lateral divisions of the OFC are also distinctive in terms of their macro-scale connectivity to other regions of the brain. For example, the classic atlas of Walker (1940) based on the parcellation of the orbital and medial prefrontal cortex in the macaque monkey, identifies a mOFC region with connectivity to the hippocampus and associated areas of the cingulate, the anterior thalamus, retrosplenial and entorhinal cortices, and various parts of the hypothalamus (as reviewed in Elliott et al., 2000; Kringelbach and Rolls, 2004; Henssen et al., 2016). In contrast, the lOFC can be further subdivided into three sectors, each having long-range anterior-posterior connectivity absent of distinct anatomical boundaries. Specifically, the anterior portion is characterized by connections to the dorsolateral prefrontal cortex (dlPFC), the insula, the mediodorsal thalamus and the inferior parietal lobule, while the caudal portion of the lOFC is characterized by heavy connections with the midline thalamus, the amygdala, and the temporal pole (as reviewed in Elliott et al., 2000; Kringelbach and Rolls, 2004). More recently, studies drawing upon the *in vivo* technique of resting-state functional magnetic resonance imaging (fMRI) concur in parcellating the OFC into medial and lateral subregions, based on whole-brain functional connectivity (e.g., Kahnt et al., 2012; see also Figure 2).

As the function of any given neuron within the central nervous system depends on its pattern of connectivity to the other neurons in the brain, the distinctive connectivity patterns of the mOFC and lOFC can be interpreted as indications that these two subregions serve distinctive functions. Thus, in our consideration of the cortico-striatal-thalamic loop circuits of the OFC, we will examine separately the structure and function of the OFC’s medial and the lateral divisions.

### Structural Connectivity of Medial and Lateral OFC Cortico-Striatal Circuits

There are distinct structural connectivity patterns for the medial and lateral portions of the OFC. The medial orbitofrontal cortico-striatal loop originates in the mOFC, consisting of the orbital gyrus and gyrus rectus and extending laterally until the medial orbital sulcus (Mega et al., 1997; Chiavaras et al., 2001). The mOFC then projects to the ventromedial caudate (VM Caudate), the ventral putamen, and the medial portion of the nucleus accumbens (NAcc; Haber, 2003; Bonelli and Cummings, 2007; Jarbo and Verstynen, 2015). From these areas, the circuit projects to the ventral pallidum and substantia nigra (SN; Cummings, 1993) before continuing to the dorsomedial, ventroanterior and ventrolateral nuclei of the thalamus (Carpenter et al., 1976; Nauta, 1979; Elliott et al., 2000). The loop is then closed with projections from these thalamic regions returning to the mOFC (Ray and Price, 1993; Figure 1A). In addition to this cortical-striatal circuit, the mOFC has strong reciprocal connectivity to many other cortical and subcortical limbic regions (Price, 2007). Notably, the mOFC shares reciprocal connections with the basolateral amygdala (Mega et al., 1997), the anterior cingulate cortex (ACC; Pandya et al., 1981), the hippocampus, the posterior parahippocampal cortex (Cavada et al., 2000). Additionally, there are strong cortico-cortical connections between the posterior cingulate cortex (PCC) and the mOFC (Cavada et al., 2000).

The lateral orbitofrontal loop, on the other hand, originates in the lOFC, which consists of the pars orbitalis region of the inferior frontal gyrus as well as the lateral, anterior and posterior orbital gyri, extending medially until the medial orbital sulcus (Chiavaras et al., 2001; Tekin and Cummings, 2002; Barbas, 2007). From here, the lOFC sends projections to the VM Caudate, which then connects to the medial portion of the globus pallidus interna (GPi) and the SN (Szabo, 1962; Johnson and Rosvold, 1971; Nauta, 1979). These regions then project to the dorsomedial and ventroanterior nuclei of the thalamus (Carpenter et al., 1976; Selemon and Goldman-Rakic, 1985) before closing the circuit with collections from the thalamic nuclei to the lOFC (Ilinsky et al., 1985; Ray and Price, 1993; Figure 1B). Additionally, the lOFC shares strong connections with the sensory regions of the inferior temporal cortex (Martin-Elkins and Horel, 1992), frontal operculum (Hackett et al., 1998), and insular cortex (Cavada et al., 2000), as well with premotor areas (Bates and Goldman-Rakic, 1993; Morecraft and Van Hoesen, 1993).

### Functional Connectivity of Medial and Lateral Subdivisions of the Orbitofrontal Cortex

It is becoming increasingly evident that many psychiatric disorders arise due to dysfunction not in isolated brain regions but rather across large-scale brain networks (Menon, 2011; Kaiser et al., 2015; Coutinho et al., 2016; Downar et al., 2016). Moreover, numerous therapeutic neurostimulation techniques used for the treatment of these psychiatric diseases, such as rTMS and DBS, selectively target functional networks to exert a therapeutic effect (Fox et al., 2014). Thus, in addition to understanding the anatomical structure of the mOFC and lOFC loops, it is also critical to examine any differences that arise in the functional connectivity to the mOFC and lOFC.

Drawing upon functional connectivity data obtained in the resting state, using *K*-means clustering, Kahnt et al. (2012) found a stable two-cluster solution of the OFC, consisting of medial and lateral subregions that corresponded well to those that had been previously defined from structural anatomical data (Figure 2A). Similarly, drawing upon task-based activations, and using predefined medial and lateral seeds, Zald et al. (2014) utilized meta-analytic connectivity modeling (MACM) to determine regions that were functionally connected to the subregions of the OFC based on patterns of co-activation during task performance. The mOFC was found to coactivate with areas of the default mode network (DMN) including the ventromedial prefrontal cortex (VMPFC) and the PCC. Additionally, the mOFC was functionally connected to the subgenual and pregenual cingulate cortices, the VM Caudate, the ventral striatum, and limbic areas comprising the amygdala, hypothalamus, and hippocampus (Zald et al., 2014). In contrast, the lOFC coactivated with cognitive control regions including the dlPFC and dorsomedial prefrontal cortex (dmPFC), the bilateral anterior insula, and the rostral ACC (rACC) extending to the pre-supplementary motor area (pre-SMA).

In terms of subcortical coactivations, in the MACM analysis above, the lOFC was shown to be functionally connected to the striatum, the bilateral thalamus, and medial temporal lobe (MTL) regions including the bilateral amygdala and left hippocampus. There was also strong functional connectivity observed between the lOFC and the fusiform gyri, the lateral occipital cortex, and the left superior temporal gyrus (STG). There was surprisingly little overlap apparent between the functional connectivity profiles of the mOFC and lOFC, suggesting once again that these subregions are not only anatomically, but functionally distinct (Zald et al., 2014; Figure 2B). Similar patterns of connectivity for the medial and lateral portions as described in the above MACM analysis were also found in the resting state, when resting-state functional connectivity (rsFC) was examined from seeds matching the previously mentioned *K* = 2 cluster solution of the OFC (Kahnt et al., 2012; Figure 2C). Thus, the available evidence from functional connectivity studies, whether on task or at rest, suggests that the mOFC and lOFC have distinct positions and roles within the overall functional architecture of the human brain. Let us now turn to examine these distinct functions in greater detail.

### Functional Roles of the Medial and Lateral OFC Cortico-Striatal Loops

In recent years, there has been tremendous progress in our understanding of the functional roles that the OFC plays in a wide variety of complex human behaviors. The OFC is an important node of multiple networks involving both visceral and motor systems; it is thought to serve as a nexus for sensory integration, particularly in the context of value-guided behavior (Kringelbach and Rolls, 2004). However, concerning the functions of the subregions of the OFC, several parallel, yet complementary, schools of thought have developed. These include, but are not limited to, reward and reversal learning (Kringelbach, 2005; Fellows, 2007), the assignment of value (Montague and Berns, 2002), reward prediction error and fictive error (Boorman et al., 2009, 2013), the generation of affective states (Bechara et al., 2000), emotional reevaluation and reappraisal (Johnstone et al., 2007; Wager et al., 2008), and decision making (McClure et al., 2004). Here, we will review how these different functions are mediated by the OFC, with particular focus on any distinct contributions that the medial and lateral portions may have.

Considerable focus has been devoted to elucidating the relationship between the OFC and reward and reversal learning. With respect to reward-guided learning, the mOFC is thought to encode the relative value of rewarding stimuli, and to learn based on probabilistic feedback (Kringelbach and Rolls, 2004; Kringelbach, 2005). For instance, the mOFC has been shown to represent the hedonic value of a stimulus independently of its identity and intensity (Kringelbach and Rolls, 2004). Similarly, there is a positive association between mOFC activition and the degree to which an individual’s decision are rational and uninfluenced by irrelevant features of the context in which they are made (De Martino et al., 2006). Interestingly, the importance of the mOFC for reward learning has been further corroborated by animal lesion studies, where lesions to the mOFC impaired an animal’s ability to associate a previously non-rewarded stimulus with reward (Jones and Mishkin, 1972; Noonan et al., 2010). In terms of learning from probabilistic feedback, Dalton et al. (2016) demonstrated that inactivation of the mOFC (via microinfusion of GABA_A_ and GABA_B_ agonists) in a rodent model induced selective impairments in probabilistic learning as indexed by reduced sensitivity to positive and negative feedback. Finally, there is a growing body of evidence that implicates the mOFC in reward learning in the context of the storage of option values (Kable and Glimcher, 2009). Thus, the mOFC appears to be necessary for encoding subjective stimulus value and for learning from probabilistic feedback on the rewarding attributes of a particular stimulus during reward-guided learning.

**Figure 3:**
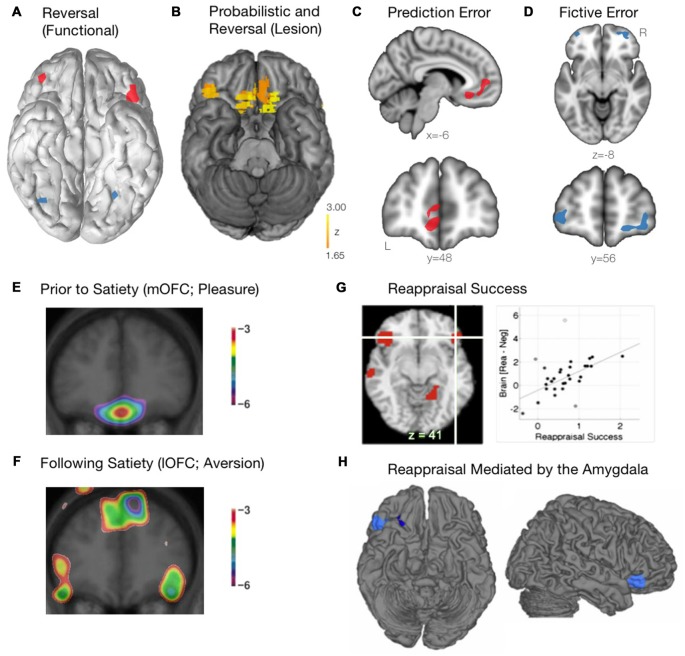
**Functional roles of the mOFC and lOFC subregions. (A)** The lOFC is selectively activated during reversal learning, when a behavioral switch was required. Schematic adapted from Kringelbach and Rolls (2004). **(B)** The critical role of the right lOFC and the mOFC for probabilistic and reversal learning, as determined using voxel-based lesion-symptom mapping in patients with focal lesions of the OFC. Adapted from Tsuchida et al. (2010). **(C)** The mOFC encodes prediction error, calculating the difference between the actual and expected reward outcome. Schematic adapted from Boorman et al. (2009). **(D)** The lOFC encodes fictive error, computing the evidence for switching behavior based on reward value of previously unchosen actions. Schematic adapted from Boorman et al. (2009). **(E)** When a stimulus (eating chocolate) is rated as being highly pleasurable prior to satiety, the mOFC is recruited. Courtesy of Small et al. (2001). **(F)** As participants continued to eat the chocolate beyond satiety, the lOFC becomes preferentially activated. Courtesy of Small et al. (2001). **(G)** Successful emotional reappraisal is correlated with activation of the lOFC. Adapted from Wager et al. (2008). **(H)** During successful emotional reappraisal, the right lOFC is mediated by the amygdala. Adapted from Wager et al. (2008).

The lOFC, on the other hand, is crucial for reversal learning, which involves adapting behavior to favor a previously unrewarded stimulus (Clark et al., 2004; Fellows, 2007). Using functional neuroimaging (fMRI), Kringelbach and Rolls (2004) demonstrated that the lOFC was selectively activated during reversal learning, when a behavioral switch between two different stimuli was required (Figure 3A). Additionally, in patients with lesions in differing regions of the OFC, those with bilateral lesions to the anterolateral OFC, but not the mOFC, were severely impaired on a visual discrimination reversal task (Hornak et al., 2002). Furthermore, Tsuchida et al. (2010) used a technique known as voxel-based lesion-symptom mapping in patients with focal lesions to the frontal lobes to determine which regions were critical for performance on a probabilistic learning task involving reversal. The authors found that lesions specifically within the mOFC and right lOFC were driving the poor task performance (Figure 3B). While the contributions of the mOFC and lOFC to task performance were not examined separately, it might be assumed that selective lesions to the mOFC were inhibiting probabilistic learning, while the lesions in the right lOFC were driving the inability to properly use reversal learning. Finally, in preclinical studies, lOFC lesions have resulted in an animal’s inability to inhibit a response to a stimulus that was previously rewarding (Jones and Mishkin, 1972), while also dramatically impairing reversal learning ability (Izquierdo et al., 2004; Noonan et al., 2010; Dalton et al., 2016). Thus, while the mOFC is responsible for encoding the relative value of a stimulus, the lOFC has a critical role in reversal learning and adapting behavior based on the most rewarding outcome.

An emerging and complementary field of study with respect to the role of the OFC and reward-based decision-making has focused on the separation between reward prediction error and fictive error. Prediction error, an important aspect of value-based learning, is calculated as the difference between the actual reward outcome and the expected outcome (Keiflin and Janak, 2015). Using a simple decision-making task, Boorman et al. (2009) used a Bayesian reinforcement learning model to demonstrate that the mOFC tracked the prediction error signal during the decision making process (Figure 3C). The mOFC, therefore, tracks the relative advantage of sticking with the current behavior (Boorman et al., 2009). Alternatively, the lOFC was found to track counterfactual incentive signals (i.e., the discrepancy between a “fictive” outcome, one that has not been experienced, and an actual outcome). By tracking this reward fictive error signal (Figure 3D), the lOFC is able to calculate the relative advantage that one would incur by switching behavior over sticking with the current behavior (Boorman et al., 2009).

Apart from reward-based decision making, the OFC also plays a critical role in both stimulus reappraisal and emotional reevaluation and reappraisal. In order to determine how a stimulus reappraisal is mediated within the frontal lobes (i.e., a stimulus that is initially judged as being positive is later judged as being negative), Small et al. (2001) fed chocolate to participants until satiety while they underwent a positron emission tomography (PET) scan. Initially, when the stimulus was rated as being highly pleasurable (and thus a hedonic stimulus), the mOFC was recruited as expected (Figure 3E). As participants continued to eat the chocolate beyond satiety, however, it was the lOFC that was preferentially activated (Figure 3F). Thus, a differential pattern of activity throughout the OFC is observed as a previously rewarding (appetitive) stimulus is reappraised to be aversive. These findings further corroborate the notion that the neural representation of reward (appetitive stimulus; mOFC) and punishment (aversive stimulus; lOFC) may involve separate motivational systems. It should be noted, however, that the replicability of these findings remains unclear. That multiple factors may play a role is in line with the finding that the specificity of “reward” and “punishment” representations within the OFC has been inconsistent, found in some instances (O’Doherty et al., 2001; Small et al., 2001; Hornak et al., 2002; Cheng et al., 2016), but not others (Small et al., 2003). Such discrepancies may, in part, be due to procedural and technical differences across studies (i.e., behavioral task, neuroimaging modality and parameters). Furthermore, it is often necessary during emotion regulation to cognitively reappraise a situation or an emotional stimulus. Indeed, using fMRI, Wager et al. (2008) demonstrated that activation of the lOFC was tightly associated with reappraisal success when participants were forced to cognitively reappraise an aversive image (Figure 3G). Additionally, when examining regions related to reappraisal success that were mediated by the amygdala (where BOLD activity within the amygdala was found to drive subsequent reappraisal by other brain regions), the rLOFC was found to be most closely linked to emotion regulation (Figure 3H; Wager et al., 2008). Within the context of decision making, the OFC has been shown to play a critical role in controlling the transition between goal-directed behavior and habitual behavior following the revaluation of various outcomes. Gremel and Costa (2013) demonstrated that, in a rodent model, goal-directed behavior was associated with increased engagement of the OFC when neuronal activity was measured *in vivo*. In addition, increasing activation of the OFC optogenetically led to an increase in goal-directed behavior relative to habitual behavior, and chemogenetic inhibition of the OFC projection neurons led to a decrease in goal-directed behavior relative to habitual behavior. Importantly, it was found that, following outcome revaluation, neurons within the lOFC were most responsible for the transition of behavior from habitual to goal-directed (Gremel and Costa, 2013).

One possible way of integrating these theories would be within the broader framework of assigning value to stimuli. In overview, the structural connections of the OFC enable the comparison of the homeostatic properties of a given stimulus against the current needs of the organism, which in turn allows for the current value of the stimulus to be determined. The value may be assigned based on the present, internal, homeostatic needs of the organism in the present moment; however, this value may also be modulated or reversed based on the current context. Medial and lateral territories of the OFC may play a role in assigning value to stimuli and in modifying or reversing this base value depending on the current context. The broader function of evaluation may provide a context in which to better understand the several different literatures that exist on OFC function in the healthy brain. They may also provide a context for better understanding the role of abnormal OFC function in psychiatric illness.

## Abnormalities of OFC-Striatal Function in Psychiatric Illness

### Major Depressive Disorder (MDD)

MDD is a common and often disabling psychiatric disorder. Although core symptoms include persistent low mood and/or a loss of pleasure or interest in previously enjoyed activities (American Psychiatric Association, 2013), the presentation of illness across individuals is quite heterogeneous. MDD symptomatology has been formulated in the RDoC framework as involving disruption across at least three domains: impulsivity and cognitive control; blunted reward learning and positive valence, and enhanced negative valence; and pathological ruminative behavior (Snyder, 2013; Goldstein and Klein, 2014; Pizzagalli, 2014). Blunted reward learning (anhedonia), present in approximately 50% of cases (Fawcett et al., 1983; Pelizza et al., 2012), can be seen as playing a critical role in the marked functional impairment that characterizes so many individuals with MDD. Moreover, poor response to antidepressant pharmacotherapy (Keedwell et al., 2005; McMakin et al., 2012; Uher et al., 2012) and neurostimulation (Downar et al., 2014) has been observed in MDD patients who present primarily as being highly anhedonic. Current treatments may thus fail to adequately address motivational and reward-processing deficits within this subgroup of patients (Price et al., 2009; McCabe et al., 2010).

Efforts to find more effective treatments that target specific symptom clusters have focused on identifying the various neurobiological pathways involved with each cluster. Neuroimaging studies have demonstrated that, compared with control subjects, those with MDD have abnormally high levels of activity in the mOFC and VMPFC (Baxter et al., 1989; Drevets et al., 1992; Biver et al., 1994; Galynker et al., 1998; Mayberg et al., 2005; Nofzinger et al., 2005; Greicius et al., 2007). In addition, several lines of evidence emerging from functional neuroimaging studies in MDD have implicated dysregulation of brain networks formed by reward-related regions such as the mOFC/VMPFC and their respective projections to the ventral striatum, amygdala, and hypothalamus. For instance, one fMRI study showed that MDD was associated with reduced corticostriatal functional connectivity between the mOFC and the dorsal ACC (dACC), precuneus, and the cerebellum (Frodl et al., 2010). Those with MDD also had diminished BOLD activation in the mOFC following the presentation of emotional stimuli (Lawrence et al., 2004), mirrored by a congruent reduction in fMRI activity in the OFC and ventral striatal regions during unexpected reward receipt (Segarra et al., 2016), and during the anticipation of monetary rewards (Smoski et al., 2011). Interestingly, other fMRI investigations have yielded divergent results, with MDD patients showing reduced ventral striatal and mOFC/VMPFC inactivation in response to pleasant stimuli and heightened activation in the caudate in response to pleasant stimuli (McCabe et al., 2009). Similar findings have also been observed in fMRI studies of remitted MDD, where patients showed reward network hyperactivation and hypoactivation during reward anticipation and reward outcomes, respectively (Dichter et al., 2012). Structurally, both the OFC and the tightly functionally connected sgACC have been shown to have significantly reduced volumes in unmedicated MDD patients compared to MDD patients receiving pharmacotherapy (Bora et al., 2012). Similarly, meta-analyses have demonstrated significant reductions in gray matter in the OFC and associated subcortical structures including the ventral striatum and amygdala in MDD (Koolschijn et al., 2009; Bora et al., 2012). Given the role of the OFC in coding both hedonic responses to reinforcers and their behavioral consequences, these results suggest that attenuated OFC function during reward outcomes may reflect diminished tagging of normally rewarding stimuli with positive affective value.

Studies investigating the disruption of “brain causal connectivity networks” in MDD have demonstrated significantly decreased Granger causality (GC; a method for detecting causal interactions between distinct brain regions) in the OFC and the caudate nucleus (Gao et al., 2016). Interestingly, incoming GC from the insula and middle/STG to the caudate nucleus were *negatively* correlated with depression severity (Gao et al., 2016). Conversely, outgoing GC values from the OFC to the ACC and occipital cortices were* positively* correlated with symptom severity (Gao et al., 2016). In addition, *changes* in reward-circuit connectivity are associated with better treatment outcomes. Consistently, recovery from depression has been associated with a decrease in mOFC/VMPFC activity (Mayberg et al., 2000, 2005; Brody et al., 2001; Dunlop et al., 2016b). In the same vein, mOFC/VMPFC lesions have been shown to confer resistance to depression (Koenigs et al., 2008), while also reducing responsiveness to negative stimuli (Damasio et al., 1990). In contrast, other functional investigations have shown that in response to positive stimuli, anhedonia symptomatology, but not depression severity, was associated with increased BOLD activity in the mOFC and decreased activity in the amygdala/ventral striatum (Keedwell et al., 2005). Dysfunction of the mOFC and VMPFC, in combination with amygdala hypoactivity, may therefore contribute to the negative symptoms of MDD such as anhedonia, amotivation, and a bias towards negatively valenced stimuli.

Dysfunction of the lOFC corticostriatal loop has also been implicated in MDD. The lOFC is prominently active during the cognitive reappraisal of emotional stimuli (Wager et al., 2008) and the tracking of counterfactual incentive signals (reward fictive error; Boorman et al., 2009). Of note, hyperactivity of the lOFC has been linked to compulsive psychopathology across multiple psychiatric disorders, including MDD (Harrison et al., 2009; Figee et al., 2013; Montigny et al., 2013; Godier and Park, 2014; Dunlop et al., 2015; Foerde et al., 2015). Indeed, increased resting cerebral blood flow (rCBF) in the lOFC has been observed in unmedicated MDD patients, with decreased metabolism being seen in these regions following antidepressant treatment (Drevets, 2007). However, the relationship between symptom severity and rCBF within these regions remains unclear, as hypermetabolic activity of the lOFC has been shown in treatment-responsive MDD patients, whereas reduced metabolic activity of the same region was observed in patients with treatment-refractory depression (Mayberg et al., 1994; Ketter and Drevets, 2002). The OFC, having direct projections to the amygdala and the hypothalamus, plays a prominent role in modulating behavioral and visceral responses to aversive stimuli. Taken together, the above findings (i.e., a negative relationship between lOFC activity and symptom severity) suggest that activation of these regions during a major depressive episode may, at least in part, function as a compensatory response for attenuating negative emotional reactivity. Furthermore, a recent study by Jollant et al. (2010), found decreased BOLD activation of the lOFC during risky choices in individuals with suicidal behavior. This finding is consistent with the suggestion that lOFC dysfunction is associated with altered processing of risk under conditions of uncertainty; further elucidating the role of the lOFC in decision-making and psychopathology. More recently, convergent evidence from neuroimaging, neurophysiological, and lesion studies, has suggested a critical role for the lOFC in a “non-reward system” (Rolls, 2016); that is, a system that responds to non-reward during reward reversal, and subsequently maintains a memory of that non-reward. However, as previously noted, aberrant orbitofrontal-striatal activity during reversal learning has been implicated in MDD (Robinson et al., 2012) as indexed by deficits in behavioral switching after reversal of the associations. Drawing upon this earlier work, Rolls (2016) recently proposed that in MDD, this lOFC non-reward system is more easily triggered, based on the premise that the omission and/or termination of a reward can propagate depression. Further evidence suggests a subsequent shift from lOFC activation to other cortical systems modulated by top-down attentional control, thereby biasing the lOFC non-reward system toward negative cognitive states (Rolls, 2013).

Further evidence for the dissociable functions of networks involving the mOFC (reward network) vs. lOFC (non-reward network) comes from a recent rs-fMRI study in a large cohort of 421 MDD patients and 488 matched healthy controls (Cheng et al., 2016). Specifically, results showed reduced functional connectivity within the medial division of the orbitofrontal circuit in MDD patients, which was negatively correlated with symptom severity (Cheng et al., 2016). As noted at the outset, the mOFC/VMPFC has been implicated in assessing the rewarding potential of subjectively pleasant stimuli. Depressive symptomology (e.g., anhedonia), may therefore, at least in part, be mediated by weakened functional connectivity between “reward-related” brain areas (i.e., mOFC) and “memory” areas (i.e., the parahippocampal gyrus and the MTL; Cheng et al., 2016). Interestingly, the opposite pattern was observed for neural circuitry involving the lOFC, with MDD patients showing *increased* functional connectivity between the lOFC and the precuneus, the angular gyrus, and the temporal visual cortex (Cheng et al., 2016). Weaker functional connectivity between these regions was associated with an increase in illness duration. These brain regions have also been associated with the subjective experience of agency and language processing, respectively, which has led to the hypothesis that dysfunction of the lateral orbitofrontal “non-reward” circuit may lead to the generation of negative self-thoughts and reduced self-esteem; two important factors in the development and maintenance of MDD (Wegener et al., 2015). Taken together, these findings provide evidence for the notion that disturbances in the functional balance between the mOFC and lOFC, and their associated corticostriatal circuit loops, contribute to specific symptom clusters in MDD.

### Obsessive Compulsive Disorder (OCD)

OCD is a disabling and difficult-to-treat neuropsychiatric illness characterized by the presence of intrusive, repetitive and unwanted thoughts (obsessions) and repetitive, ritualistic behaviors (compulsions; Wood and Ahmari, 2015). Given its chronic nature, OCD is a leading cause of illness-related disability, with up to 2%–3% prevalence worldwide (Koran, 2000). Despite the high prevalence and considerable socioeconomic burden of OCD, progress in understanding this illness and in developing effective treatments remains limited. Some individuals improve on pharmacotherapy, with the most commonly used agents being selective serotonin reuptake inhibitors (SSRIs) or clomipramine (a tricyclic antidepressant with particularly strong serotonergic activity) and antipsychotics (D2 receptor antagonists). Structured psychotherapeutic interventions such as cognitive behavioral therapy (CBT) are also helpful in some individuals. However, the majority of OCD cases do not achieve remission on current treatments, and a substantial fraction experiences no improvement at all (Eisen et al., 1999; Bloch et al., 2013). Consequently, efforts are underway to better understand the pathophysiology of OCD and to develop novel interventions that target this pathophysiology directly.

The literature linking OCD to abnormal OFC cortico-striatal-thalamo-cortical activity is extensive and extends back several decades (reviewed in Menzies et al., 2008). Early proposals for basal ganglia dysfunction as an underlying cause of OCD were published in the 1980s, with PET neuroimaging demonstrating metabolic abnormalities in OFC and associated striatal regions around the same time (Baxter et al., 1988, 1992). Several excellent reviews have recently been published on the role of corticostriatal circuits through the OFC and their role in OCD, and the reader is encouraged to refer to these for a comprehensive exploration of this topic (Menzies et al., 2008; Del Casale et al., 2011; Milad and Rauch, 2012). It is important to note that the literature implicates a number of corticostriatal pathways in OCD pathology other than the OFC—most prominently, pathways through the dACC and dmPFC (Radua et al., 2010), and pathways from the amygdala to orbital and medial prefrontal cortex (Milad and Rauch, 2012). Here, in keeping with the topic of this review, we will focus specifically on abnormalities in orbitofrontal corticostriatal circuits.

Structural neuroimaging studies in OCD have been sufficiently numerous to enable several quantitative meta-analyses in recent years. Meta-analyses of voxel-based morphometry (VBM) studies in OCD patients reveal increases in gray matter volume in the head of the caudate nucleus and in the neighboring, slightly posterior regions of the putamen and globus pallidus (Radua and Mataix-Cols, 2009). Other studies and meta-analyses have also reported increases in OFC gray matter in OCD (e.g., Valente et al., 2005; Christian et al., 2008; Rotge et al., 2010; Togao et al., 2010). Increases in white matter volume have also been reported for the anterior limb of the internal capsule and orbitofrontal region (Togao et al., 2010). However, one study found that symptom severity was correlated to a decrease in gray matter in the lOFC specifically (Koprivová et al., 2009).

Of note, lesion studies also implicate OFC corticostriatal circuits in OCD pathology. “Secondary OCD” can result from lesions of the basal ganglia (Carmin et al., 2002), particularly in the head of the caudate (Chacko et al., 2000), or from infarct or injury to the left or right OFC (Kim and Lee, 2002; Ogai et al., 2005). Conversely, there are also striking cases in which patients with longstanding, intractable OCD have showed marked *improvement* in symptoms following basal ganglia hemeorrhage affecting the OFC-striatal circuit (Yaryura-Tobias and Neziroglu, 2003; Fujii et al., 2005).

Functional imaging studies in OCD have employed PET, SPECT, and fMRI techniques. As noted above, early PET studies indicated increased metabolic activity in the subcortical components of the OFC-striatal circuitry, particularly in the head of the caudate nucleus; an associated early finding was that successful treatment with SSRIs or CBT reduced the hyperactivity in this region (Baxter et al., 1992). fMRI studies have provided further refinements by demonstrating that the functional connectivity between the ventral striatum and other prefrontal regions, including the OFC, is increased in OCD patients (Sakai et al., 2011). Seed-based analyses have found that OCD patients have greater connectivity from ventral striatum to mOFC, but lower connectivity from dorsal putamen to lOFC, compared to controls (Harrison et al., 2009). Studies applying mathematical technique of GC (which can establish directional relationships of influence between regions, as opposed to the non-directional associations of simple correlation) have demonstrated that orbitofrontal regions have elevated causal influence over the activity of the ventral striatum in OCD patients (Abe et al., 2015). In addition, fMRI studies have found increased whole-brain functional connectivity for the subthalamic nucleus and the mOFC and lOFC in OCD patients (Beucke et al., 2013). Overall illness severity has been reported to correlate to the functional connectivity between ventral caudate regions and the mOFC and lOFC (Harrison et al., 2013). Finally, fMRI studies have shown that successful treatment with brain stimulation interventions such as rTMS or DBS appears to reduce the functional hyperconnectivity between prefrontal cortical regions and the ventral striatum/head of the caudate nucleus (Figee et al., 2013; Dunlop et al., 2016b), thus providing evidence that the hyperconnectivity in these pathways is not merely epiphenomenal but may in fact have a causal role in perpetuating the illness.

Emerging evidence has implicated the OFC (particularly the lOFC) in switching between habitual behavior and goal-directed behavior following outcome evaluation (Gremel and Costa, 2013). Given that OCD is characterized by unwanted, intrusive thoughts (obsessions) and repetitive, stereotyped behaviors (compulsions), it is possible that many features of the disorder arise due to an inability to transition from habitual to goal-directed behavior. Indeed, Gillan et al. (2011) found that OCD patients had deficient action control during a goal-directed learning task, often relying on habits. Additionally, symptom provocation in OCD patients led to an increase in activation of brain regions associated with habitual behaviors and a decrease in activation of regions associated with goal-directed behaviors (Banca et al., 2015).

As a summary of the state of the literature to date, a recently published meta-analysis of structural and functional neuroimaging studies in OCD examined data from VBM studies enrolling 928 patients vs. 942 controls, and fMRI studies of inhibitory control enrolling 287 patients and 284 controls (Norman et al., 2016). In the VBM meta-analysis, OCD patients showed widespread decreases in gray matter volume in mOFC, ACC and dmPFC, and DLPFC; lateral orbitofrontal regions were conspicuously absent from the findings. Increases in gray matter volume were reported bilaterally in the NAcc, putamen, and globus pallidus as well as left caudate nucleus. In the fMRI meta-analysis, OCD patients performing inhibitory control tasks (e.g., Stroop, Flanker, or Go/No-Go) showed less activation in regions including the ACC and dmPFC and right caudate nucleus, but greater activation in regions including the putamen and ventrolateral prefrontal cortex. Although these findings confirm the important role of frontal-striatal circuits in OCD pathophysiology, they also indicate that the set of frontostriatal circuits affected by OCD extends beyond simply the OFC-striatal loop circuit itself. In particular, loop circuits serving the ACC and dmPFC appear to be affected. Furthermore, the findings support previous work in suggesting that the mOFC and lOFC-striatal circuits may be affected differentially in OCD, with lOFC loop circuits showing signs of hypertrophy and hyperactivity, and mOFC circuits showing signs of atrophy and hypoactivity.

### Substance Use Disorders (SUDs)

SUDs, characterized by excessive and compulsive intake of drugs of abuse, were once thought to be solely reliant upon the mesolimbic dopamine system due to its central role in the brain’s reward circuitry (Wise, 1996; Leshner, 1997; Schoenbaum and Shaham, 2008). There are, however, multiple hallmark features of SUDs that cannot be explained solely in terms of reward-system dysfunction, and are better characterized by compulsivity (a lack of control over drug intake or compulsive drug use). For instance, those with SUDs disregard the negative consequences associated with acquiring and taking the drug, give up previously enjoyed activities in favor of drug use, expend inordinate time and effort to obtain the drug of abuse, become preoccupied with the drug, and continue to administer the drug even in the absence of a pleasurable response (Fischman et al., 1985; Volkow and Fowler, 2000; Coffey et al., 2003). In addition, even if prolonged abstinence from drug use is attained, relapse is common (Shalev et al., 2002; Epstein et al., 2006). Thus, due to their role in mediating goal-directed behavior, both mOFC reward and lOFC compulsivity mechanisms may be required to explaining how problems with higher level cognitive functioning arise in SUDs.

The OFC has been repeatedly implicated across a variety of SUDs. Structurally, the OFC has shown decreased gray matter density in those addicted to cocaine in comparison to controls (Franklin et al., 2002; Matochik et al., 2003; Ersche et al., 2011; Smith et al., 2015), and smaller volume in chronic alcohol abusers (Laakso et al., 2002; Thayer et al., 2016) Additionally, altered metabolism of the OFC has been observed across numerous SUDs. Decreased resting activity of the OFC is reliably observed during late withdrawal (abstinence for 7 days or more) for methamphetamine (London et al., 2000, London et al., 2004; Volkow et al., 2001; Sekine et al., 2003), cocaine (Adinoff et al., 2003), alcohol (Dao-Castellana et al., 1998), and poly-substance abusers (Stapleton et al., 1995). During short-term withdrawal (abstinence for less than 7 days), however, increased metabolism has been shown for abusers of methamphetamine (London et al., 2004) and cocaine (Volkow et al., 1991). In addition to structural and functional abnormalities within the OFC, those with SUDs perform poorly on the Gambling Task (Grant et al., 2000; Bolla et al., 2003; Dom et al., 2005), a test of real-world decision-making known to rely heavily on the OFC (Bechara et al., 1999; Li et al., 2010). While it is clear that SUDs are associated with dysfunction of the OFC, it is important to examine how the medial and lateral portions may differentially contribute to specific symptomatology observed.

The mOFC is tightly connected to limbic areas (Pandya et al., 1981; Mega et al., 1997; Rolls, 2015) and it plays a role in the active monitoring and evaluation of competing reward related stimuli (Rushworth et al., 2011). Therefore, it might be expected that its dysfunction in SUDs be related to any emotional response associated with the drug, craving, sensitization, and difficulties with prediction error. Indeed, Goldstein et al. (2007) demonstrated that, compared to controls, cocaine-addicted subjects showed greater hypoactivation of the mOFC and greater distractibility when viewing drug related stimuli during a drug Stroop fMRI task, suggesting an inability to suppress the emotional intensity and task-irrelevant emotional information (Goldstein et al., 2007). Also, the mOFC was shown to have heightened sensitivity to the administration of a stimulant (methylphenidate) in a cocaine-addicted population when compared to controls, and the observed increases in mOFC activity were associated with mood elevation in the cocaine group (Volkow et al., 2005). This increased mOFC sensitivity may directly contribute to the increased emotional reactivity to the drug during the development of craving in SUDs. In fact, inducing craving causes an increase in OFC metabolism (Wang et al., 1999), and the successful control of craving is associated with a decrease in the metabolism of the mOFC (Volkow et al., 2010). An important aspect of reward guided learning carried out by the mOFC is prediction error, where the difference between the actual and expected reward outcome is calculated (Keiflin and Janak, 2015). It has been shown that those with SUDs are less sensitive to loss and have impaired tracking of prediction error in the mOFC (Tanabe et al., 2013). Additionally, a decreased neural response to negative prediction error is observed in the addicted population suggesting that reward guided learning from failure is blunted (Parvaz et al., 2015). Finally, those with SUDs were shown to have decreased gray matter volume in the mOFC which was significantly correlated with levels of risk-taking behavior during a decision making task (Tanabe et al., 2013). Taken together, these studies provide evidence for abnormal activity of the mOFC promoting heightened emotional responses to drugs and drug stimuli, craving and becoming preoccupied with acquiring and self-administering the drug even in the face of negative consequences.

On another view, the lOFC monitors and compares the sensory environment encoding alternative courses of action, and modifies behavior according to the most rewarding outcome (Rushworth et al., 2011). Abnormal functioning of the lOFC may therefore be responsible for the deficient action planning and context-induced drug-seeking behavior associated with SUDs. An important function of the lOFC is the coding of fictive error, or the evidence for switching behavior based on the reward value of previously unchosen actions (Boorman et al., 2009). Chiu et al. (2008) demonstrated that while a fictive error signal is computed in smokers, it is later ignored, providing evidence for how problems with action planning appear in those with SUDs. In addition, ignoring the fictive error signal may result in the choice of immediate reward over delayed gratification, which has been observed in a population of cocaine-addicted patients (Coffey et al., 2003). Furthermore, Kufahl et al. (2008) showed that unexpected, but not expected, cocaine infusion resulted in an activation of the lOFC. Such findings suggest that the disruption of lOFC functioning in SUDs may mediate faulty behavioral planning and the effects of contextual factors on drug use.

Taken together, several lines of evidence have implicated orbitofrontal-striatal dysfunction in the pathophysiology of numerous psychiatric disorders (Harrison et al., 2009; Figee et al., 2013; Montigny et al., 2013; Godier and Park, 2014; Dunlop et al., 2015; Foerde et al., 2015), including those reviewed here. Although the primary symptom and neuropsychological profiles of MDD, OCD, and SUDs differ, common orbitofrontal-striatal disturbances have been observed across these disorders. For example, structural neuroimaging studies investigating MDD, OCD, and SUDs have demonstrated decreased GMV in the OFC and associated subcortical structures, including the ventral striatum and amygdala (Franklin et al., 2002; Matochik et al., 2003; Koolschijn et al., 2009; Radua and Mataix-Cols, 2009; Ersche et al., 2011; Bora et al., 2012; Smith et al., 2015). Also, there appears to be common functional aberrations involving orbitofrontal-striatal circuitry among these disorders; although, neurobiological distinctions between them are insufficiently clear as comparative neuroimaging studies are scarce. For example, several functional imaging studies have indicated hypoactivity of the mOFC pathway in OCD (Norman et al., 2016) and in SUDs (Goldstein et al., 2007), but not in MDD (Baxter et al., 1989; Drevets et al., 1992; Biver et al., 1994; Galynker et al., 1998; Mayberg et al., 2005; Nofzinger et al., 2005; Greicius et al., 2007); however, other lines of investigation have shown a functional disassociation between the mOFC and lOFC, with hyperactivity and hypoactivity of these pathways being observed in some cases (Harrison et al., 2009). Conversely, MDD has been associated with abnormally high levels of mOFC activity, with reduced FC between this region and its subcortical components of the mOFC-striatal circuit (Baxter et al., 1989; Drevets et al., 1992; Biver et al., 1994; Galynker et al., 1998; Mayberg et al., 2005; Nofzinger et al., 2005; Greicius et al., 2007).

Although converging evidence suggests that orbitofrontal-striatal dysfunction characterizes MDD, OCD and SUDs, how these aberrations translate into the affective, cognitive, and behavioral manifestations of each disorder remains to be fully elucidated. As noted at the outset, current conceptions propose that the lOFC is crucial for the flexible control of behavior, response inhibition, self-control, and reversal learning. This link between lOFC function and the suppression of behaviors/emotions is consistent with current evidence implicating aberrant lOFC-striatal activity with compulsive psychopathology across MDD, OCD, and SUDs. The mOFC-striatal loop, on the other hand, is critical for assigning value to stimuli based on the internal homeostatic needs of the individual. Aberrant functioning of this circuit may thus lead to inappropriate value assignment for stimuli causing the emergence of select psychiatric symptomatology such as anhedonia in MDD and OCD, and cravings in SUDs.

## Therapeutic Mechanisms of Neurostimulation Techniques Targeting The OFC Corticostriatal Circuits

### Deep Brain Stimulation (DBS)

DBS is an invasive form of neuromodulation that involves the stereotaxic implantation of one or more electrodes into a target region of the brain. Differing stimulation parameters are used to either increase (excitatory) or decrease (inhibitory) activity of that region, while at the same time causing widespread changes in activity throughout the associated cortical-subcortical loops (Lakhan and Callaway, 2010; Holtzheimer and Mayberg, 2011). DBS techniques targeting the mOFC and lOFC circuits have been used to treat a variety of psychiatric disorders, including MDD, OCD, SUDs and eating disorders.

For MDD, inhibitory stimulation of the sgACC has been repeatedly shown to have antidepressant efficacy (Mayberg et al., 2005; Lozano et al., 2008; Puigdemont et al., 2012). Interestingly, sgACC-DBS also has a strong anti-anxiety effect and is associated with a decrease in activity of the mOFC (Lozano et al., 2008). DBS of the sgACC has also shown efficacy in the treatment of anorexia, increasing BMI from baseline and elevating mood (Lipsman et al., 2013).

Alternatively, excitatory DBS of the NAcc in patients with MDD has been demonstrated to have, in addition to a robust antidepressant effect, both strong anti-anxiety and anti-anhedonic effects, which are correlated with decreased metabolic activity in both the mOFC and the lOFC (Schlaepfer et al., 2008; Bewernick et al., 2010). For the treatment of OCD, NAcc-DBS leads to reductions in obsessive-compulsive behavior and anxiety symptoms, while at the same time increasing libido (Denys et al., 2010). Although OCD is typically thought of as an anxiety disorder, the presence of anhedonic symptomatology, separate from that explained by comorbid depression, has been observed in patients with OCD (Abramovitch et al., 2014). Thus, it is possible that by targeting the NAcc with DBS in OCD patients, a similar pattern of activation changes as Bewernick et al. (2010) reported in depressed patients would be observed.

SUDs are associated with dysfunction of the reward circuitry (Leshner, 1997). Targeting the reward circuitry, whether it be cortically or subcortically, may therefore result in a decrease in addictive symptoms. Both Kuhn et al. (2009) and Mantione et al. (2010) observed a serendipitous cessation of smoking in patients receiving NAcc-DBS for OCD. In addition, Müller et al. (2009) reported a case where stimulation of the NAcc was successfully able to treat chronic resistant alcohol abuse.

Stimulation of the ventral capsule/ventral striatum has also been shown to decrease depressive symptomatology in those with MDD (Malone et al., 2009), and DBS of the STN (Mallet et al., 2008) and the ventral caudate (Aouizerate et al., 2004) led to remission from OCD symptoms in some cases.

### Repetitive Transcranial Magnetic Stimulation (rTMS)

rTMS is a form of non-invasive neuromodulation that employs a handheld induction coil placed against the scalp in order to apply focused magnetic field pulses to target brain regions (Hallett, 2007). Inhibitory forms of rTMS, thought to act via long-term depression, include low frequency (1 Hz) stimulation (Chen et al., 1997) and continuous theta burst stimulation (cTBS; Huang et al., 2009). Excitatory forms on the other hand, thought to act via long-term potentiation, include high frequency (5–20 Hz) stimulation (Pascual-Leone et al., 1994) and intermittent theta burst stimulation (iTBS; Huang et al., 2005). Individual variability of response, however, remains a current drawback to all forms of rTMS—certain individuals show increased synaptic plasticity to inhibitory forms of stimulation, and vice versa (Maeda et al., 2000; Eldaief et al., 2011).

Although the OFC is implicated across a wide range of psychiatric conditions, a limited number of studies have specifically targeted this region with rTMS. The therapeutic use of rTMS has been best characterized for the treatment of MDD, with most studies choosing to target the dlPFC (Berlim et al., 2014; Kedzior et al., 2014; De Raedt et al., 2015) and more recently the dmPFC (Bakker et al., 2015; Dunlop et al., 2015). Those studies that have targeted the mOFC and lOFC, however, have had promising results for the use of rTMS in psychiatric disorders characterized by aberrant OFC activity.

**Figure 4:**
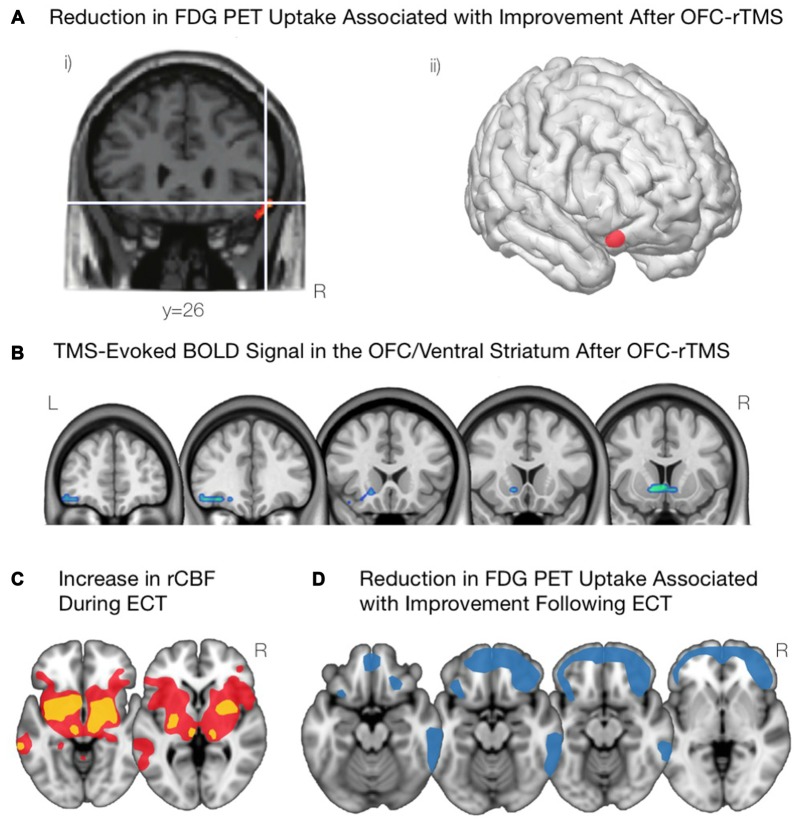
**Modulations of OFC activity associated with non-invasive brain stimulation paradigms. (A)** Following inhibitory OFC-repetitive transcranial magnetic stimulation (rTMS), obsessive compulsive disorder (OCD) symptom improvement was associated with a reduction in activity of the right lOFC, as shown in (i) and visualized in (ii). Adapted from Nauczyciel et al. (2014). **(B)** Inhibiting the mOFC with rTMS in cocaine addicted subjects led to an attenuation of craving that was associated with decreases in activation of the lOFC and the mOFC. Courtesy of Hanlon et al. (2015). **(C)** Acute administration of electroconvulsive therapy (ECT) reveals increases in regional cerebral blood flow in the lOFC and associated striatal components including anterior striatum, amygdala, globus pallidus, and thalamus. Yellow indicates areas with a greater increase in regional cerebral blood flow; red indicates areas with a lower increase in regional cerebral blood flow. Schematic adapted from Takano et al. (2007). **(D)** Depressive symptom improvement following ECT is correlated with reductions in activity of the bilateral OFC and the frontal pole. Schematic adapted from Henry et al. (2001).

In OCD patients, the application of inhibitory (1 Hz) rTMS to the left (Ruffini et al., 2009) and right (Nauczyciel et al., 2014) lOFC has significantly reduced levels of obsessive-compulsive behavior. In addition, the decrease in OCD symptomatology was significantly correlated with a decrease in metabolism of the lOFC (Figure 4A; Nauczyciel et al., 2014). In the context of SUDs, since the control of craving in cocaine-addicted subjects was associated with a decrease in activation of the mOFC (Volkow et al., 2010), it would stand to reason that inhibiting this region with rTMS would produce a similar result. Indeed, inhibiting the left mOFC with cTBS in those addicted to cocaine led to an attenuation of craving that was associated with a decrease in activation of the lOFC and mOFC (Figure 4B; Hanlon et al., 2015). Taken together, these results suggest that rTMS may be able to effectively and selectively modulate psychiatric symptomatology in which the OFC is implicated.

### Electroconvulsive Therapy

ECT, one of the oldest and most widely used forms of neuromodulation, involves the induction of therapeutic seizures by passing a train of electrical current pulses through the brain via electrodes placed either unilaterally or bilaterally on the scalp over the frontal lobes or temporal lobes. Several meta-analyses have demonstrated the efficacy of ECT in the treatment of both MDD (unipolar depression) and bipolar depression, with remission rates exceeding 50% even in treatment-refractory cases (Kho et al., 2003; Pagnin et al., 2004; Dierckx et al., 2012). For certain types of depression, such as psychotic depression, ECT may achieve remission rates of up to 90% (Petrides et al., 2001). However, in other populations, such as borderline personality disorder, outcomes for ECT are substantially less impressive, with remission rates as low as 20% (Feske et al., 2004). Understanding the mechanisms of ECT, and why they are so effective for some patients but not others, is therefore a priority.

Neuroimaging studies over the last 20 years have provided some evidence on the mechanisms of ECT, and how they may alleviate depression symptoms. PET scans have been acquired during the acute administration of ECT, and reveal that the acute stimulation triggers marked increases in activity subcortically, appearing as increases in regional cerebral blood flow throughout the anterior striatum, globus pallidus, thalamus, amygdala, and brainstem as well as in the lOFC (Takano et al., 2007; Figure 4C). PET scans acquired a few days after ECT reveal marked decreases in metabolic activity throughout the medial and lateral prefrontal cortex (Nobler et al., 2001). However, the degree of *improvement* post-ECT appears to correlate more specifically to reduction in metabolic activity in the OFC and nearby frontopolar cortex (Henry et al., 2001; Segawa et al., 2006; Figure 4D).

Structural neuroimaging studies have also examined neural predictors and correlates of ECT outcome. In a recent VBM study (Wade et al., 2016), MDD patients showed lower volumes in NAcc and adjacent globus pallidus compared to controls. After ECT, patients showed overall increases in the volume of the left putamen, and ECT responders showed increases in the volume of the NAcc compared to non-responders. These findings indicate that the therapeutic mechanisms of ECT are apparent not only as changes in brain function, but also as changes in brain structure; moreover, therapeutic response involves changes in the subcortical components of the OFC-striatal loop circuits.

Similar conclusions emerge from other recent studies using functional MRI to examine how ECT affects functional connectivity in MDD (Leaver et al., 2016). Compared to healthy controls, patients with MDD showed baseline hyperconnectivity between the ventral striatum and ventral regions of the default-mode network, within the region of the mOFC, as well as reciprocal pattern of hypoconnectivity from ventral striatum to more anterior regions of the default-mode network. ECT successfully normalized (i.e., reduced) the hyperconnectivity from ventral striatum to ventral default-mode regions, without affecting the hypoconnectivity from ventral striatum to more dorsal regions. As these more dorsal regions have been linked to cognitive control and impulsivity (e.g., as reviewed in Dunlop et al., 2016a), these findings suggest an explanation for why ECT may offer lesser benefit in MDD patients with comorbid deficits in cognitive control or impulse regulation, such as those with borderline personality disorder (Feske et al., 2004).

### Transcranial Direct Current Stimulation (tDCS)

tDCS, like rTMS, is a non-invasive neuromodulation technique that is thought to act via modulation of the synaptic plasticity of target brain regions and their respective subcortical loops (Brunoni et al., 2012; Dunlop et al., 2016a). Constant current low-energy stimulation (1–2 mA) is applied to the brain over a montage of two or more scalp electrodes, and a typical session of tDCS lasts from 5 to 30 min. As a therapeutic intervention, the duration of a typical course of tDCS consists of daily stimulation for 10–30 days (Meron et al., 2015). Cathodal stimulation is typically considered to be inhibitory (leading to a decrease in synaptic plasticity), while anodal stimulation is considered to be excitatory (leading to an increase in synaptic plasticity). Similar to rTMS, the individual variability of response to tDCS remains a drawback for this form of neuromodulation (Wiethoff et al., 2014).

The majority of the studies examining the therapeutic efficacy of tDCS have employed excitatory (anodal) stimulation to the left dlPFC for the treatment of MDD and SUDs (Brunoni et al., 2012; Kekic et al., 2016). Relatively few studies, however, have investigated tDCS targeting the OFC. Given the benefits of inhibitory rTMS of the OFC in patients with OCD, it is possible that this lower-energy form of neuromodulation may lead to similar outcomes when applied in an inhibitory fashion. Mondino et al. (2015) administered 10 twice-daily sessions of inhibitory (cathodal) tDCS of the left OFC in a case of treatment resistant OCD. A decrease in obsessive-compulsive behavior was observed following the treatment course that was maintained at a 1-month follow-up (Mondino et al., 2015). Using the same protocol in an open-label, uncontrolled study, Bation et al. (2016) also reported a significant and lasting decrease in OCD symptomatology. Interestingly, however, no decrease in depressive symptoms was observed (Bation et al., 2016).

Study of the therapeutic mechanisms of tDCS at the neural level is still in its infancy. So far, it remains an unresolved question as to whether the mild electrical currents of tDCS are sufficient to exert an effect on subcortical structures, such as the basal ganglia. Some initial work suggests that this may be possible. For example, resting-state fMRI studies of tDCS stimulation of the primary motor cortex have found that a session of stimulation increased the functional connectivity from primary motor cortex to thalamus (Polanía et al., 2012). Another study using the functional neuroimaging technique of arterial spin labeling (ASL) found that tDCS to lateral prefrontal regions can decrease resting perfusion of the head of the caudate nucleus; this same study found that tDCS is also capable of modulating the activity of both the mOFC and the lOFC (Weber et al., 2014). In addition, a recent study demonstrated that anodal tDCS of the frontal pole (with the cathode over dlPFC) was able to modulate the activity of a mOFC circuit extending into the ventral tegmental area during the viewing of attractive faces; moreover, faces were perceived as more attractive during such stimulation (Chib et al., 2013). Such results indicate that tDCS targeting the mOFC or lOFC is feasible, that such stimulation may engage the cortico-striatal-thalamo-cortical circuits serving these target areas, and that such stimulation may exert effects on reward functions. All of these findings are encouraging for future therapeutic applications of tDCS to treat illnesses characterized by OFC-striatal pathology.

## Unresolved Questions and Future Directions

### Contributions of Medial and Lateral OFC Circuits to Psychiatric Illness

Although it is reasonably well established that the mOFC and lOFC (and their associated corticostriatal loop circuits) have distinct contributions to brain function, it is still an unresolved issue as to how these distinct subregions contribute to the pathophysiology of the major categories of psychiatric disease. Recent literature suggests that these pathways may indeed contribute to distinct symptom clusters within a given disorder. For example, in MDD, abnormal function and connectivity in the mOFC pathway has been linked to deficiencies in hedonic capacity and reward learning (e.g., Smoski et al., 2011; Segarra et al., 2016). Conversely, abnormal function and connectivity in a lOFC “non-reward” pathway has also been recently reported, and potentially linked to a sensitivity to negative affective stimuli and a propensity toward negatively valenced ruminations (e.g., Cheng et al., 2016; Rolls, 2016). Likewise in OCD, distinctive patterns of hypoconnectivity through mOFC-striatal pathways and hyperconnectivity through lOFC-striatal pathways has been reported; however, contrary reports are also to be found in the literature, and some authors have proposed a more nuanced model of lOFC vs. mOFC function in OCD (e.g., Milad and Rauch, 2012). More broadly, mOFC and lOFC-striatal loops have been linked to transdiagnostic constructs such as the RDoC domains of “positive valence systems” and “negative valence systems”, respectively (e.g., Webb et al., 2016). Such constructs, grounded in neuroanatomy, may provide an alternative formulation of symptomatology in psychiatric illness (e.g., Dunlop et al., 2016a) as their distinctive roles in psychopathology are clarified.

### Relative Contributions of OFC vs. Non-OFC Loop Circuits to Psychopathology

Another important question concerns how the pathology arising from mOFC- and lOFC-striatal dysfunction can be distinguished from, or related to, pathology arising from dysfunction in other cortico-striatal circuits outside the OFC. For example, in OCD, it is now recognized that non-OFC pathways are consistently implicated in the disorder (Milad and Rauch, 2012), with ACC and dorsomedial pathways showing structural and functional abnormalities that are if anything more consistently observed than those in the OFC (e.g., Norman et al., 2016). Likewise for MDD and SUD, circuits such as the “salience network” (SN; comprising ACC and insular regions, as well as dorsolateral prefrontal and inferior parietal regions) are recognized to play an important role in cognitive control, with the SN acting as an “anti-network” in opposition to the mOFC reward circuit (Downar et al., 2016; Dunlop et al., 2016b). Indeed, deficits in SN structure and function also appear transdiagnostically in psychiatric illnesses (Goodkind et al., 2015). Such deficits may underlie pervasive deficits in cognitive control (another RDoC domain) seen in many Axis I disorders (McTeague et al., 2016). An important question for future study will thus be how dysfunction of the SN and other non-OFC pathways contributes to the major categories of psychiatric illnesses, and whether SN dysfunction combines with mOFC or lOFC dysfunction to generate psychiatric symptomatology.

### Neurally-Defined Subtypes/Endophenotypes of Axis I Disorders

As the relationship between specific corticostriatal pathways and associated brain functions becomes clearer, it becomes possible to envision a “neuroanatomical formulation” of the specific constellation of symptoms presenting in individual patients with MDD, OCD, SUD, or other psychiatric illnesses. For example, within the heterogeneous category of MDD patients as defined by DSM-V criteria, there exist dimensions of illness along which any given individual patient may be positioned: e.g., high disruption of cognitive control, relative preservation of reward sensitivity, and high non-reward predisposition/neuroticism. An “RDoC Formulation” approach to psychiatric illness is very much in its infancy; however, if these symptom dimensions can be reliably linked to discrete neural pathways, then a neuroanatomical formulation of individual patients with MDD, SUD, OCD, or other disorders may gradually become feasible. Indeed, recent literature has already begun to parse the heterogeneity of DSM disorders such as MDD into neural dimensions or “neural endophenotypes” in this manner (e.g., Webb et al., 2016). In the past, a neuroanatomical formulation would have been of limited use, given that available interventions (psychotherapy, pharmacotherapy) were not very anatomically specific in their effects. However, with the emergence of a suite of more anatomically precise neurostimulation interventions (DBS, rTMS and tDCS, among others), it may become not only feasible but critical to tailor the stimulation target to the neuroanatomical site of dysfunction. At present, there are no established, reliable methods in routine clinical use for tailoring the neurostimulation target or parameters to the individual patient based on symptoms, testing, or imaging. However, recent advances in this area suggest that this approach is likely to gain in feasibility and importance in the near future (Drysdale et al., 2017).

### Feasibility of Selective, Non-Invasive Stimulation of Medial and Lateral OFC Circuits

As noted above, neuroanatomical formulation of psychiatric illness becomes important only when the set of available interventions can be directed precisely and selectively against the dysfunctional circuit of interest. Thus, as we gain a better understanding of the distinctive contributions of mOFC- and lOFC-striatal loop circuits to the pathology of MDD, OCD and other psychiatric illnesses, it will become increasingly important to determine how selectively we can stimulate these pathways. For invasive techniques such as DBS, selective stimulation may be relatively straightforward, and indeed judicious placement of electrode contacts within the internal capsule, ventral striatum, or other subcortical regions may even allow simultaneous and independent modulation of the mOFC vs. lOFC pathways. For non-invasive techniques such as rTMS and tDCS, it remains to be seen how selectively these circuits can be targeted. For rTMS, fMRI studies to date suggest that frontopolar stimulation may be capable of targeting mOFC-ventral striatal pathways (Hanlon et al., 2013). Similarly, PET studies suggest that stimulation of the lOFC may be possible, and tolerable, in patient populations (Nauczyciel et al., 2014). For tDCS, fewer studies are available, but preliminary evidence suggests that stimulation of mOFC-subcortical reward pathways and lOFC-striatal pathways may be possible (Chib et al., 2013; Weber et al., 2014). Whether tDCS can target these regions selectively remains to be established. In general, further studies are needed to establish whether selective non-invasive stimulation of mOFC and lOFC pathways is feasible, and if so, what effects ensue, behaviorally and clinically, from stimulation of each target.

### Efficacy of Novel Interventions Targeting Medial and Lateral OFC Circuits

As much as preclinical literature points to the importance of mOFC and lOFC-striatal dysfunction in psychiatric illness, from a translational point of view, the key question is whether interventions targeting the OFC actually show therapeutic efficacy in treating psychiatric disorders. On this topic, the presently available literature is encouraging, but still embryonic. To date, OFC-rTMS and OFC-tDCS have been applied in small, preliminary studies in patients with OCD, showing some promising effects (Nauczyciel et al., 2014; Mondino et al., 2015; Bation et al., 2016). Preclinical studies have also targeted the OFC in SUD with single-session rTMS (e.g., Hanlon et al., 2016); however, full therapeutic courses of treatment at this target have not yet been reported. A single case report is also available showing successful treatment of MDD with low-frequency right OFC-rTMS (Fettes et al., 2017). In this latter case, an encouraging feature is that the patient had previously failed to respond to rTMS of two more conventional targets (the DMPFC and DLPFC). Thus an important question for future study is not merely whether therapeutic courses of OFC-rTMS and OFC-tDCS show efficacy vs. sham, but also whether they show efficacy in individuals who do not respond to stimulation at other targets. If so, then the proportion of patients who can achieve remission via non-invasive brain stimulation may increase substantially.

## Conclusion

In this review article, we have surveyed a substantial and fast-growing literature indicating that OFC-striatal circuits have important roles to play in valuation, affect regulation and decision-making. Furthermore, we have reviewed a growing body of evidence that dysfunction in these OFC-striatal circuits may be central to the pathophysiology of a variety of psychiatric illnesses. Although the role of these pathways in OCD has been recognized for decades, OFC-striatal dysfunction now appears to play an important role in the pathophysiology of MDD, SUD, and potentially many other major categories of psychiatric disease. Dysfunction in mOFC or lOFC-striatal pathways is amenable to intervention by brain stimulation. Although invasive techniques such as DBS can target the relevant pathways directly, non-invasive techniques also appear capable of modulating the activity of OFC-striatal loops. Such modulation may be central to the therapeutic mechanisms of well-established techniques such as ECT, as well as newer techniques such as rTMS and tDCS. At present, these latter techniques are only just beginning to be applied to the OFC, but the small number of preclinical studies and pilot trials conducted to date appear promising in their findings. For the many patients in whom existing treatments have failed, OFC-striatal interventions may have the potential to succeed.

## Author Contributions

PF, LS, and JD were responsible for writing sections of manuscript. PF was responsible for creating all figures.

## Funding

PF has received funding from the Canadian Institute for Health Research (CIHR) Canadian Graduate Scholarship and the Ontario Government via the Ontario Graduate Scholarship. LS has received support from the Ontario Brain Institute via the Canadian Biomarker Initiative in Depression (CAN-BIND). She is also a recipient of the Ontario Mental Health Foundation (OMHF) studentship. JD has received research support from CIHR, the Klarman Family Foundation, the Buchan Family Foundation, and the Toronto General and Western Hospital Foundation. He has also received a travel stipend from Lundbeck and from ANT Neuro, and in-kind equipment support for an investigator-initiated study from Tonika/Magventure.

## Conflict of Interest Statement

The authors declare that the research was conducted in the absence of any commercial or financial relationships that could be construed as a potential conflict of interest.
